# How to effectively promote interprofessional collaboration? – a qualitative study on physicians’ and pharmacists’ perspectives driven by the theory of planned behavior

**DOI:** 10.1186/s12913-021-06903-5

**Published:** 2021-09-02

**Authors:** Łucja Zielińska-Tomczak, Magdalena Cerbin-Koczorowska, Piotr Przymuszała, Ryszard Marciniak

**Affiliations:** Department of Medical Education, Poznan University of Medical Sciences, 7 Rokietnicka St, 60-806 Poznan, Poland

**Keywords:** Interprofessional care, The theory of planned behavior, Pharmacist-physician collaboration, Pharmaceutical care, Healthcare professionals’ attitudes

## Abstract

**Background:**

Ajzen’s theory of planned behavior (TPB) postulates that individuals’ behavioral intention is influenced by their attitudes, subjective norms, and perceived behavioral control. Therefore, it can be used to broaden the understanding of particular behaviors, including healthcare workers’ professional activities.

**Methods:**

In this study, we used TPB as a theoretical framework to evaluate semi-structured interviews with pharmacists and physicians to build an understanding of the interprofessional collaboration between them. Sixteen semi-structured interviews were conducted with pharmacists and eleven with physicians. The sample of participants comprised a diverse group with varying work experience and workplaces. Data were analyzed independently by two researchers following the thematic analysis method using ATLAS.ti software. Data saturation was set in the absence of new issues arising during the interviews.

**Results:**

The content analysis allowed for the determination of six main themes: the relationship between previous experiences and attitudes towards collaboration, pharmacist’s role in collaboration, mutual reluctance toward collaboration, the role of decision- and policy-makers, knowledge and qualifications gaps regarding collaboration, and lack of organizational paths.

**Conclusions:**

Despite both physicians and pharmacists displaying positive attitudes towards collaboration may foster their intention to establish a professional partnership, subjective norms (e.g., the lack of appropriate legal regulations) and perceived behavioral control (physicians’ lack of awareness about pharmacists’ qualifications and the low level of interpersonal skills) might impede the process.

## Introduction

The collaboration of healthcare professionals allows for the provision of more comprehensive care to the patients, which contributes to improved quality of treatment, reduced incidence of medical malpractice, shortened hospitalization, and lower mortality rate [1]. As a result, the popularity and importance of interprofessional care (IPC) are increasing [2]. The emphasis is to establish a patient-centered partnership between different healthcare team members and take advantage of their combined knowledge and skills for patient care improvement [2].

In the opinion of the World Health Organization experts, IPC should be a standard practice in patient care as a critical element in ensuring the high quality of health services [1]. An example of a patient-orientated interprofessional service is pharmaceutical care, which assumes establishing collaboration between a physician and a pharmacist for ensuring a high quality of health services [3].

In many countries, the collaboration between physicians and pharmacists is formally established. For example, in the United States, collaborative practice agreements allow pharmacists to provide clinical services in collaboration with doctors, such as hypertension management programs [4]. In Australia, pharmacists, after a referral from the general practitioner, carry out Home Medicines Reviews, aiming to improve the safety and effectiveness of pharmacotherapy [5]. Furthermore, Dutch community pharmacists and physicians organize regular pharmacotherapy audit meetings in order to improve the quality of pharmacotherapy [6].

Although the European Directorate for the Quality of Medicines & Health Care emphasizes the need for governments and policy-makers to “acknowledge available evidence that the pharmaceutical care philosophy and working methods can help achieve the benefits of responsible medicine use for individual patients and healthcare systems at national and regional levels by addressing issues of inappropriate medicine use in a comprehensive manner and, thereby, improving patient outcomes” [7], the actual provision of pharmaceutical care in Europe is still limited [8]. Over 20 years ago, van Mill et al. [9] observed that the attitudes of pharmacists and other healthcare professionals were two of the ten major barriers to IPC implementation in European countries. Since then, many interventions have been developed to enhance such collaboration [10]. However, in their systematic review, Bollen et al. [10] concluded that most studies focus on the educational effectiveness of those initiatives and not the resulting behavioral changes.

## Background

Taking into account the strong influence of theory on study design [11], we chose Ajzen’s theory of planned behavior (TPB) as a theoretical framework for this study. Our decision was guided by the results of the meta-analysis performed by Armitage and Conner [12], which demonstrated the ability to predict one’s intentions and behavior. As systematic reviews suggest, the behavioral intention may also be an important indication of the behavior of healthcare professionals [13, 14].

TPB assumes that one’s intentions directly influence their behavior and are determined by attitudes, subjective norms, and perceived behavioral control [15]. Individual attitudes towards behavior are characterized by personal insights about a particular action. They present positive or negative beliefs about the activity and expectations regarding its effects. Subjective norms denote the perceived pressure of the environment and indicate an individual’s perception of what is expected from them in a particular context. Perceived behavioral control presents a subjective evaluation of the difficulty or ease of taking action. It also indicates potential factors inhibiting a particular behavior, even if the individual has positive attitudes towards it [16].

The usefulness of TPB in understanding and predicting the professional behaviors of healthcare professionals was presented previously [16–22], including studies on pharmacists using both quantitative [19–21, 23] and qualitative methodology [22]. For this reason, this study aimed to draw upon TPB to understand physicians’ and pharmacists’ intentions regarding interprofessional collaboration. Evaluating their attitudes, subjective norms, and perceived behavioral control variables will allow identifying factors crucial for developing their behavioral intention towards it [16]*.*

## Methods

The study involved face-to-face semi-structured interviews conducted from October 2018 to December 2019 with respondents from three regions of Poland (Mazovia, Greater Poland, and West Pomerania). The participants were licensed, professionally active pharmacists and physicians. According to the registers kept by Chambers of Physicians or Pharmaceutical Chambers, 150,000 physicians and 36,000 pharmacists hold licenses to practice in Poland as of 2019. The majority of Polish physicians practice in hospitals, specialist clinics, and general practice, while pharmacists primarily practice in community and hospital pharmacies [24].

Invitations to participate in the study were distributed personally in clinics and community pharmacies as well as sent electronically via social networking sites and administrative offices of Chambers of Physicians or Pharmaceutical Chambers, which distributed the invitations among their members. The invitation contained information about the aims of the project and its scientific and noncommercial character. It also assured that participation in the study was voluntary, and the respondent might reject participation or withdraw at any stage of the project. They were also informed that obtained data would only be used for scientific purposes and analyzed and presented only in anonymized form, not allowing to identify individual participants.

Interviews were conducted outside the respondent’s place and time of work to ensure freedom of expression. During the study, attention was paid to the convenience of the participants, especially in the case of respondents from other regions of Poland. Therefore the choice of time and place for the interview was dictated solely by respondents’ preferences and included, for instance, our departments’ office, a park nearby to respondents’ workplace, or a coffee shop. Before each interview, the study protocol was discussed with the participant to explain potential concerns about the study and its aims, and informed consent was obtained and recorded.

Interviews were based on a flexible thematic guide, asking the same questions to all respondents. This method allows for richer, more detailed data to be gathered compared to quantitative methods and allows the researchers to understand respondents’ attitudes, opinions, and points of view [25, 26]. The interview guideline was designed by the research team, and the outline of covered topics is presented in Table 1. The clarity of exemplar questions asked during the interviews was later verified by two pharmacists and two physicians.

**Table 1:** The semi-structured interview guideline

The topic	Sample questions
Physicians and pharmacists’ contact in professional matters.	Have you ever contacted a physician in professional matters? How did the conversation(−s) go?
Physicians and pharmacists’ collaboration in one’s near environment.	How does the collaboration between physicians and pharmacists look like in your environment?
The situation in Poland.	How do you feel about this situation in Poland?
Possibilities of collaboration.	What possibilities of collaboration do you see?
The pharmacist’s potential role in the patient care team.	What role should the pharmacist play in the patient care team?
Form of collaboration.	Where and in what form could the collaboration of doctors and pharmacists take place?
Barriers to collaboration.	What hinders building collaboration between doctors and pharmacists?
Improvement of collaboration.	How may collaboration be improved?

Interviews were conducted in Polish by the main researcher and recorded with the use of Sony ICDTX50 sound recorder. The average time of interviews was 14 min 9 s (range from 8 to 30 min). Every interview and associated demographical data of the respondent were given a code number immediately after recording to protect the anonymity of participants and further processed and analyzed only in this coded form.

Data were analyzed independently by two researchers – ŁZT, PP (researcher triangulation), which allowed viewing the results from a broader perspective [27]. Data analysis was carried out using ATLAS.ti software and followed the thematic analysis method described by Braun and Clark [28]. Data saturation was set when no new issues arose during the interviews and occurred in thirteen interviews with pharmacists and seven with physicians.

All methods were performed following the relevant guidelines and standards for reporting qualitative research developed by O’Brien et al. [29]. The study’s project was presented to the Bioethical Committee of the Poznan University of Medical Sciences, which confirmed that its approval was not required according to the Polish law and guidelines provided on the Committee’s website [30]. Despite the lack of need for approval from the Bioethical Committee, great efforts were made to ensure the ethical course of the study. At every stage, care was taken to protect the anonymity of the respondents and the confidentiality of obtained results. Before participating in the study, the informed consent of the interviewees was also obtained. In the invitation to the study, all potential respondents were informed about its purpose and course, including the audio recording of interviews, the voluntary character of participation, the possibility of resigning from participation at any stage of the project, and principles of data collection and use. Finally, we made efforts to minimize any potential inconveniences for respondents resulting from their participation in the study. Therefore, they could choose the most optimal time and place to give an interview.

## Results

In response to the invitations, thirty-three replies from pharmacists and physicians were received. In six cases, the potential respondent’s participation in the study was not possible due to scheduling difficulties despite repeated attempts or the objection to recording the interviews. The sample comprised of a diverse array of individuals from a variety of workplaces and with distinct work experiences (Table 2). In total, sixteen semi-structured interviews were conducted with pharmacists and eleven with physicians. In the case of pharmacists, eleven of them worked in community pharmacies, with the remaining working in hospital pharmacies. Three of them worked in rural areas and thirteen in urban areas. Three respondents from the group of pharmacists had a specialization. Out of the eleven physicians interviewed by us, seven worked in a hospital, three in a local medical center, and one in a specialized private office. Five of them had a specialization, five were pursuing specialization, and one neither had a specialization nor was pursuing one.

**Table 2:** The characteristics of respondents

Code	Workplace (type/localization)	Specialization	Seniority (years)
Pharm 1	Community pharmacy/ big town	None	4
Pharm 2	Community pharmacy/ medium town	None	4
Pharm 3	Community pharmacy/ small town	None	3
Pharm 4	Hospital pharmacy/ big town	clinical pharmacy	30
Pharm 5	Community pharmacy/ big town	None	1
Pharm 6	Hospital pharmacy/ big town	None	1
Pharm 7	Community pharmacy/ big town	during specialization	2
Pharm 8	Community pharmacy/ big town	None	2
Pharm 9	Community pharmacy/ village	None	17
Pharm 10	Hospital pharmacy/ big town	None	2
Pharm 11	Community pharmacy/ big town	None	2
Pharm 12	Community pharmacy/ big town	None	18
Pharm 13	Hospital pharmacy/ big town	hospital pharmacy	12
Pharm 14	Hospital pharmacy/ medium town	None	4
Pharm 15	Community pharmacy/ village	community pharmacy	30
Pharm 16	Community pharmacy/ village	None	3
Doc 1	Hospital/ big town	during specialization	3
Doc 2	Hospital/ big town	during specialization	3
Doc 3	Hospital/ big town	during specialization	3
Doc 4	Hospital/ big town	Specialist	10
Doc 5	Hospital/ big town	Specialist	14
Doc 6	Hospital/ big town	during specialization	4
Doc 7	Hospital/ big town	resident	4
Doc 8	General practice/ small town	none	3
Doc 9	Specialist clinic/ village	specialist	11
Doc 10	General practice/ village and big town	specialist	40
Doc 11	General practice/ village	specialist	52

The characteristics of respondents’ localization* were prepared on the basis of data from the Polish Central Statistical Office; small towns - population below 20,000 inhabitants, medium towns - population 20,000–100,000 inhabitants, large towns - population above 100,000 inhabitants [31].

**Fig. 1:**
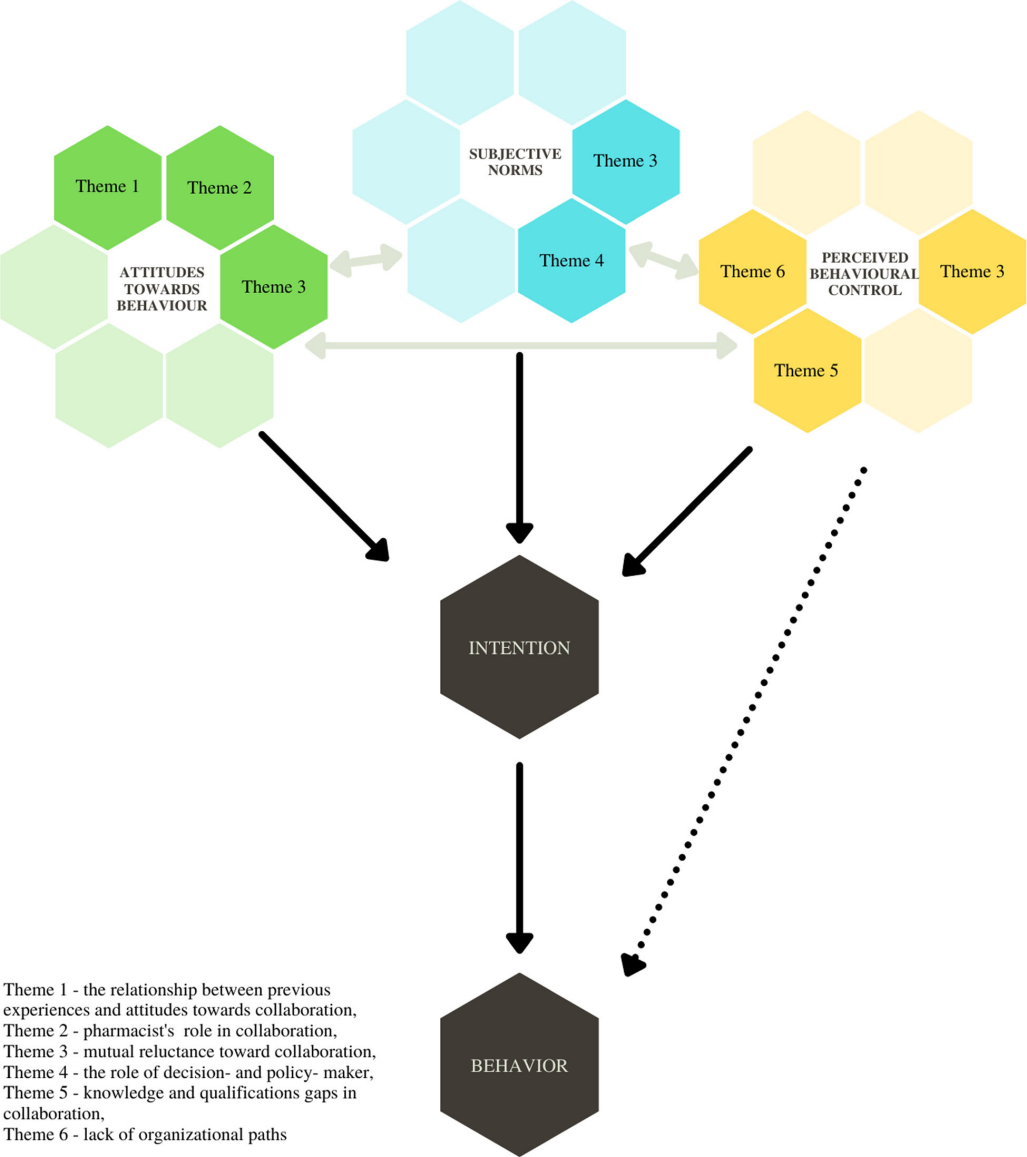
A construct model of Ajzen’s theory of planned behavior, integrated with the results of the study

The content analysis allowed determining six main themes: the relationship between previous experiences and attitudes towards collaboration, pharmacist’s role in collaboration, mutual reluctance toward collaboration, the role of decision- and policy-makers, knowledge and qualifications gaps in collaboration, and lack of organizational paths. The topics identified cover all constructs described by TPB, as presented in Fig. 1.

### Theme 1: The relationship between previous experiences and attitudes towards collaboration

The nature of previous experiences with collaboration between physicians and pharmacists appeared to affect their attitudes toward establishing a partnership in the future. While most of these experiences were usually based on contacts related to technical and formal matters (which may diminish their interest in interprofessional collaboration), some pharmacists, especially working in hospitals, report more frequent and substantive ways of collaboration. Additionally, on a more individual level, the quality of these meetings influenced future contact, as evidenced by examples of poor previous experiences translating into negative attitudes and a reluctance to contact a given doctor.

Past experiences mentioned by the respondents mainly concerned formal aspects of patient care such as prescription writing, realizing orders, drug availability, or drug substitution. Although described as occurring less frequently, the scope of substantive consultations included preparing prescription drugs or selecting over-the-counter medicaments.

*Pharm 2: “Contact with doctors, it was mainly to consult dosage, whether it is really what we see on the prescription or the doctor made a mistake.”*

*Doc. 11: “I call the pharmacy to ask which medicines were withdrawn and which are available.”*

*Doc 5: “Most often, it involves correcting my prescription errors.”*

A slightly broader picture of collaboration emerges from the statements depicting the clinical environment.

*Pharm 13: „We also run a Cytostatic Drug Lab and Parenteral Nutrition Lab, and in that scope, contacts with doctors are permanent.”*

The significance of past experiences appears to significantly influence the views of pharmacists and physicians on further collaboration. As one respondent noted on the example of prescription errors and how negative experiences instilled a desire to avoid them in the future:

*Pharm 1: “If a patient comes with a prescription from a doctor who poorly conducted such a conversation in the past, I send him back [to the doctor].”*

### Theme 2: Pharmacist’s role in collaboration

The majority of respondents held positive beliefs about the effectiveness of establishing interprofessional collaboration between pharmacists and physicians. They also identified exemplar division of roles between the doctor and the pharmacist, which may serve as a measure of their attitudes toward behavior. Participants with a more positive view towards the broader scope of the collaboration were seemingly more inclined to give more detailed descriptions of the potential roles of a pharmacist in the interprofessional team. On the other hand, some respondents focused on the pharmacist’s role only as a physician’s assistant, which may clarify their less than favorable perceptions of broadening their scope of practice.

The form of collaboration described most often by physicians involved a pharmacist as a physician’s advisor. Potential areas for collaboration mentioned by both doctors and pharmacists were similar and included: planning and monitoring patient pharmacotherapy and patient education. In their statements, the emphasis was on the pharmacist’s role as an expert on drugs.

*Doc 4: “Medical order sheets or treatment protocols could be checked and verified by pharmacists. ( …) Pharmacists should have an advisory role as experts in pharmacokinetics, adverse drug reactions.”*

*Doc 7: “The role of pharmacists should involve educating patients on drug administration, combining drugs. ( …) It would be ideal if we could use pharmacists’ knowledge to create pharmacokinetic models in difficult cases.”*

*Pharm 1: “A pharmacist should revolve around drugs, starting from kinetics, dynamics, drug interactions.”*

*Pharm 14: “Discussing adverse effects, providing patients with information on interactions - this is a key role of a pharmacist.”*

While some pharmacists expressed similar opinions, emphasizing the importance of involvement in the design and management of the patient’s pharmacotherapy as an opportunity to use their intellectual potential, one respondent expressed a concern that the physician’s “assistant” role might lower their professional prestige.

*Doc 5: “A pharmacist could work as a pharmacotherapy consultant.”*

*Pharm 4: “Pharmacists don’t make final decisions; they are advisors.”*

*Pharm 16: “The presence of a pharmacist in the hospital ward would contribute to greater use of the pharmacist’s potential.”*

*Pharm 10: “Pharmacists should be specialists in their field, and the term ‘helper’ is too pejorative to refer to them ( …). However, as a compromise, the term ‘pharmacotherapy consultant’ has been coined. It somehow shows the merits that pharmacists may bring, but does not equate them with physicians.”*

Even those physicians who did not see the need to collaborate with a pharmacist expressed the need to seek expert advice in the field of pharmacotherapy, with particular emphasis on pharmacokinetics.

*Doc 5: “I don’t see such a need for collaboration with a pharmacist. (...) I am referring to the pharmacokinetics of drugs, and here I would miss such consultations.”*

Of particular note, only one respondent indicated that the relationship should be based on mutual benefits.

*Pharm 10: “Three areas where it overlaps. The first - synergism of helping patients, ( …) the second form of collaboration between a physician and a pharmacist is that the physician learns from the pharmacist ( …) and the other way around, the third form is what the pharmacist learns from the physician”.*

### Theme 3: Mutual reluctance toward collaboration

Participants described a significant, often self-imposed isolation between physicians and pharmacists with simultaneous reluctance to contact each other. This insolation is exacerbated by the fear of being judged by a representative of the other professional group and pharmacists’ low confidence in their competency (perceived behavioral control). Another significant barrier limiting contact is the existence of stereotypes of both professions (subjective norms). However, increased informal contact with members of the other profession allowed for steps to be made towards overcoming these stereotypes.

Respondents reported mutual antipathy and reluctance to contact the “other” profession, which may impact the very intention of undertaking interprofessional collaboration.

*Doc 7: “I observe an unexplained communicational resistance. There is some sort of animosity between the professions.”*

*Doc 4: “I have the impression that pharmacists don’t want to come to us as a professional group.”*

This observation seems to supplement the concerns expressed about being judged by members of the other professional group.

*Doc 7: “We doctors are probably afraid that pharmacists will judge our knowledge.”*

*Doc 7: “Doctors are reluctant to use the knowledge of other health professionals.”*

Respondents also emphasize that pharmacists do not have faith in their abilities, which may cause their reluctance to establish professional relations with doctors. Stereotypes encountered in society were also seen as a significant barrier.

*Doc 4: “Students already start their education with developed stereotypes on different professional groups, and this is a barrier in making contact with other professions.”*

Pharmacists indicated that the younger generation of physicians seems more cooperative than older doctors. Physicians supported these views and suggested a positive effect of informal relationships with pharmacists on doctors’ openness towards collaboration.

*Pharm 10: “There is no denying that the younger generation of physicians is more favorable towards the collaboration of physicians with other health professions.”*

*Doc 1: “I think it depends on age and private contacts with pharmacists ( …) the younger the doctor, the more prone they are to collaborate; the more pharmacists they have in their environment, the more open they are to it.”*

### Theme 4: The role of decision- and policy-makers

Collected data illustrate that the role of decision- and policy-makers also influences the intention to undertake inter-professional cooperation. Lack of involvement from the Chamber of Physicians or Pharmaceutical Chamber and the Insurer in organizing cooperation may indicate the respondents’ perception of low social pressure towards collaboration (subjective norms).

Pharmacists emphasize the absence of legal regulations as a factor limiting the possibility of establishing partnerships with physicians. Similarly, they also noted the lack of guidelines related to collaboration, especially those that could have been prepared by the Chamber of Physicians or Pharmaceutical Chamber.

*Pharm 5: “Nobody at the level of legislation, Chamber of Physicians or Pharmaceutical Chamber has determined how this collaboration should look like.”*

Pharmacists also indicate the insurer’s (National Health Fund) role in stifling the development of collaboration. Unless therapeutic programs and medical procedures require the presence of a partnership will not be profitable.

*Pharm 6: „If the insurer doesn’t see the necessity of the presence of a pharmacist or correctly prepared pharmacy in all therapeutic programs or highly specialized medical procedures during hospitalization, these changes cannot occur. Because this kind of legislation, ordinances of the Ministry of Health or President of the National Health Fund [NFZ], it can bring evident changes in healthcare provision.”*

### Theme 5: Knowledge and qualifications gaps in collaboration

Knowledge and qualifications gaps (perceived behavioral control) may limit the intention to establish cooperation between professions. Lack of knowledge about mutual competencies and collaboration possibilities translates into a lower likelihood of contacting a representative of other medical professions. In addition, the lack of education in communication with other medical workers means that establishing contact may result in undue stress for medical professionals.

Although some pharmacists reported that they had experienced physicians’ willingness to establish interprofessional collaboration, several physicians in this study confirmed a lack of knowledge of pharmacists’ scope of expertise. This lack of knowledge results in difficulty in determining clear expectations of the collaborative roles and duties, which may constitute the reason for the lack of interest from physicians.

*Doc 4: “Physicians are afraid of collaboration because they don’t know how much they could gain from pharmacists.”*

*Pharm 11: “Doctors still don’t see that pharmacists could be of any help in their work.”*

*Doc 2: “Physicians lack knowledge on what pharmacists know. Unfortunately, in pharmacies, we often meet pharmacy technicians instead of pharmacists - it strongly affects the way physicians perceive the education of pharmacists. They do not know how much the knowledge of pharmacists differs from that of pharmacy technicians. If a doctor does not know what to ask a pharmacist, they will not ask”.*

The insufficient knowledge of pharmacists and physicians on the possibility of collaboration and the ways to start it was listed as one of the barriers.

*Pharm 10: “We are not taught how to begin [the collaboration].”*

*Doc 4: “Lack of knowledge on collaboration is a barrier. There is low awareness of the possibility of such contact and that both professional groups can gain a lot from each other.”*

*Doc 2: “I don’t know how to start the collaboration. There is no formal way to ask a pharmacist for consultation.”*

Qualification gaps were identified in the area of ​​communication with other medical professionals. Respondents mentioned both the lack of interprofessional training within the under- and post-graduate education curricula and other possibilities that would foster the formation of a relationship. As a result, in the respondents’ opinion, contact with representatives of other professions is indirectly discouraged and is a source of stress and anxiety.

*Pharm 5: “During pharmacy studies, there is a lack of classes on passing information to doctors, how to address them, how to lead the discussion.”*

*Pharm 10: “Some people never had contact with a physician or a medical student during their studies. It is surely a stressful situation when it first happens during their professional life and work.”*

*Doc 1: “Walking our educational path, we don’t cross each other, and therefore we don’t know what pharmacists can, and they don’t know what we can.”*

### Theme 6: Lack of organizational paths

Developing collaboration opportunities and organizational solutions can facilitate contact between professions and positively influence the intention of interprofessional collaboration. Conversely, their absence may serve as a barrier in acting (perceived behavioral control). Some solutions appear more formal, including hiring pharmacists in hospital departments. Unfortunately, such organizational solutions may increase the already significant amount of documentation required or negatively impact other obligations.

According to respondents, there are many opportunities to establish collaboration between both professions. However, it should be emphasized that the mentioned solutions were formal in nature, e.g., hiring pharmacists in hospital departments and including them in the therapeutic team or popularization of pharmaceutical care services. Consequently, the respondents perceived many factors discouraging them from such collaboration, for example, the additional time requirements or a significant addition to the documentation workload.

*Doc 7: “We have a lot of useless paperwork. ( …) There may be fear that yet another responsibility in the form of consultations with pharmacists would generate more paperwork.”*

*Doc 3: “I know that there is so much work in the hospital pharmacy that they don’t have time for me. If I went there, I would only extend their working day and disturb them.”*

The perception of collaboration as not an inherent element of everyday work, but rather as something more than that, makes healthcare professionals expect additional space for regular consultations and even additional remuneration.

*Pharm 1: “Pharmacies rather have as many rooms as required by law, so I think that in more than 90% of pharmacies, there won’t be a possibility to create enough space for meetings of a physician with a pharmacist.”*

*Doc 3: “There is also no place where a doctor and a pharmacist could meet because everyone is locked at their place. We lack a common space.”*

*Pharm 11: „Truth be told, it is not gratified anywhere, so if someone has a lot of determination, it can be done. But I think that no pharmacist will be tempted because it is not within the range of obligations imposed by the employer.”*

*Pharm 10: “If there were systemic solutions facilitating pharmacists’ access to the ward, their willingness would increase, I think. ( …) pharmacists with specialization in clinical pharmacy cannot work on the ward due to procedural reasons”.*

Moreover, pharmacists working in hospital pharmacies observe that their current role largely depends on the size and location of the center.

*Pharm 13: “It varies among the hospitals. Some pharmacies are only administration or storage facilities with no influence on pharmacotherapy. And contrarily, there are also pharmacies taking part in pharmacotherapy where pharmacists are engaged in the therapeutic process.”*

In some centers, a pharmacist can work with physicians as a member of thematic teams on issues such as quality of treatment, antibiotic therapy, clinical nutrition, and treatment guidelines. In smaller hospitals, contact between a doctor and a pharmacist is an essential element of work due to the lack of sufficient numbers of specialists or good quality software. The implementation of technological solutions that would facilitate the exchange of information tends to limit direct contact between specialists, favoring one-way information transfer.

*Pharm 14: “At small institutions, this is inevitable, given that the team of physicians and pharmacists is very small, such consultations are required on a daily basis to determine treatment options. At bigger centers, we have good-quality computer programs, and this collaboration is limited because we do everything using them. At small centers, there is not enough money to buy such programs. As a result, a pharmacist and a doctor have to discuss more issues to make it [pharmacotherapy] have sense.”*

*Doc. 1: “We had a program like that - our hospital pharmacy had a system called Unit Dose, and I could use it to contact a pharmacist from the hospital pharmacy. ( …) I often prescribe non-standard doses. In this program, I can contact a pharmacist to dispense a drug in starch capsules or discuss how to prepare a medication to administer the right dose.”*

## Discussion

This qualitative study examined pharmacists’ and physicians’ perceptions related to interprofessional collaboration based on variables described by Ajzen’s TPB, namely attitudes towards the behavior, subjective norms, and perceived behavioral control. Our results allowed us to characterize the way the collaboration is perceived, barriers in its implementation, and identify opportunities for change.

### Attitudes towards the behavior (ATB)

Healthcare workers’ attitudes towards certain professional behaviors may be crucial for their intention to perform the given behavior [16]. The attitudes presented by pharmacists in this study seem to confirm previous studies showing their favorability towards establishing collaboration [32–34]. Furthermore, the use of the qualitative methodology described above allowed us to explore the experiences and attitudes of respondents in a more depth manner when compared to alternative quantitative methodologies. The collected data provoke reflection on what motivates respondents to such openness in light of significant barriers to collaboration.

Respondents described the insufficient role of a pharmacist in Poland, a claim supported by the results of previous studies [35, 36]. According to Merks et al. [36], the current perception of the pharmacist profession is unsatisfactory for its representatives, who instead see themselves as knowledgeable specialists and consultants on safe pharmacotherapy. Additional studies observed that pharmacists, as well as pharmacy students, expect to broaden their professional roles in the future [37, 38].

Therefore, it seems that one of the key factors responsible for the openness of pharmacists to establish collaboration is the desire to elevate the perception of their profession. The significance of this motivation in shaping the perceived value of collaboration appears to be evidenced by concern over the perception that the pharmacist is ‘only’ a doctor’s assistant. This concern was verbalized by participants in this study. Although interprofessional collaboration would likely positively affect patient outcomes, it would not necessarily be associated with high prestige change in the perception of the pharmacist by physicians and the general public.

Joint International Pharmaceutical Federation and World Health Organization guidelines on good pharmacy practice [39] indicate that a pharmacist should be a physician’s partner in patient care, responsible for patient education and identification of symptoms. However, in view of the collected data, the question arises whether pharmacists presenting low self-confidence in their abilities will be less motivated to establish cooperation due to the fear of worsening their image.

The issue of concern over perception by the other profession is not limited to pharmacists, as indicated by the statements of the physicians interviewed in this study. One respondent admitted that they did not see the possibility of establishing collaboration with a pharmacist, despite the willingness to consult pharmacists on drug-related issues. This particular respondent described their previous experiences in the doctor-pharmacist relationship, indicating that it only involved correcting issued prescriptions. This observation seems to confirm the results of Alkhateeb et al. [40], who point out that when the relationship is based solely on formal and organizational, and not substantive aspects of pharmacotherapy, it not only does not support building an interprofessional relationship but even lowers physicians’ interest in establishing future collaboration.

Additionally, the results of this study indicate the areas that, in the opinion of representatives of both professions, could serve as a platform for establishing collaboration. Attitudes can positively affect the intention if, in the respondent’s opinion, undertaking the behavior would allow achieving the expected outcome [15]. Given the identified knowledge gaps among physicians in the area of ​​pharmacokinetics, it seems particularly justified to design a framework that would enable the use of pharmacists’ qualifications in pharmacotherapy optimization.

Considering the above, designing interventions aimed only at ATB change may have a positive effect on physicians’ intentions to undertake the behavior but not necessarily affect the intention of pharmacists due to their already strong internal motivation.

### Subjective norms (SN)

Subjective norms describe the way in which the respondent perceives the favor or lack of favor of the environment towards particular behavior [15]. In the study, a significant role of the legislators and the National Health Fund was identified in shaping the intention of undertaking collaboration by representatives of both professions. Although the current legislation indicates the need to establish such collaboration [41], in practice, it is only regulated in terms of hospital therapeutic committees [42]. The expectations of the largest Polish insurer do not go beyond the supervision of the proper trade in medicinal products [43].

Consequently, in the absence of effective legal regulations, representatives of professional circles, such as the Supreme Medical Chamber, express concerns and postulate the necessity to clarify many aspects of such collaboration, beginning with the scope and form and ending with setting standards for documentation [44]. At the same time, according to the respondents, none of the chambers have taken any steps to stimulate collaboration. In other European countries, such as Germany, experience has shown that by defending the interests of their own professional group, professional self-governments are forced to take stands that indirectly affect representatives of other professions [45].

The so-called *tribalism* can cause the fear of being criticized by other health professionals, as identified in the respondents’ statements, and be a source of mistrust towards them. Studies indicate a vital role of mutual trust in building collaboration between representatives of various medical professions, illustrating the detrimental effects of *tribalism* on collaboration [40, 46–48]. The fear of losing competence, resulting from a misunderstanding of the mutual scopes of practice and roles in patient care, contributes to the fragmentation of the healthcare services market and hinders building an integrated healthcare system [49, 50]. San Martín-Rodríguez et al. [51] underlines the importance of understanding competence interdependencies and accepting zones in which they overlap. Moreover, the fear of being deprived of competence may limit established relationships [38].

It is worth emphasizing that the awareness of such a stereotypical conflict between a physician and a pharmacist is still visible in the course of their undergraduate education, and the vast majority of last-year students of both faculties describe the quality of collaboration between professions as ‘bad’ or ‘moderate’ [38].

It seems that the attempted changes towards pharmacist’s greater involvement in the therapeutic process undermine the stereotypical perceptions of a physician as an independent decision-maker in this area [38] and a pharmacist as an individual responsible only for dispensing medicinal products, described by many researchers from various countries, including Poland [40, 52–56]. The influence of this stereotype on the perception of the possibility of establishing collaboration is also emphasized by respondents’ experiences showing a greater degree of openness towards it among younger generations. This generational change has also been reported recently by other Polish researchers [57].

To sum up, the respondents’ experiences and literature reports indicate that the possibilities provided by subjective norms in building intentions towards establishing collaboration remain underutilized. In the last 10 years, specialist literature presented some papers highlighting the importance of leadership in shaping the IPC. Authentic leadership contributes to building trust, which determines the proper functioning of inter-professional teams. It also fosters the understanding of teamwork and can contribute to developing new team roles [58, 59].

### Perceived behavioral control (PBC)

Perceived behavioral control is characterized by self-efficacy and controllability, indicating the ability and freedom to perform the behavior, respectively. In this study, the respondents’ statements illustrate their low self-efficacy for initiating interprofessional collaboration. Two elements, namely physicians’ lack of awareness about pharmacists’ qualifications and insufficient interpersonal skills, can likely attribute to this problem. The specificity of undergraduate education seems to be closely related to both of these issues. Studies conducted among students of final years in their medicine and pharmacy in Poland and the curriculum analysis in both faculties show that inter-professional relations are more often emphasized in the current pharmacy program and curriculum [38].

In the opinion of the respondents of this study, insufficient qualifications in the field of communication with representatives of other medical professions are additional sources of complications in developing IPC. The literature seems to support their claims as Luetsch and Rowett [60] noted that successful communication is the condition for IPC.

Surprisingly, even though the results of previous studies suggest a lack of appropriate teleinformatic tools dedicated to communication between specialists is a barrier to establishing partnership-based collaboration, respondents indicated that the implementation of such software might reduce the need for direct contact between them. In support of this, Sargeant et al. [61] indicate that increasing awareness and respect for roles played by other team members can be achieved by providing both formal and informal opportunities for interaction between them. Both Drovandi et al. [62] and Chui et al. [63] showed that the presence of a pharmacist in the hospital department brings benefits to patients. In these studies, interventions recommended by pharmacists were generally well accepted by physicians, and interactions between doctors and pharmacists could reveal their need for collaboration to both parties.

As for controllability, although interprofessional collaboration should be a regular element resulting from the work culture based on voluntariness [51], the participants’ responses indicate their perception of such collaboration in a far more formal light. Therefore, there is a concern that until the implementation of precise legal regulations in this area, the exchange of information regarding the patient’s health may be perceived by them as going beyond their standard competencies.

As Ries [64] points out, the law regulates the functioning and standards of the healthcare system, including the competencies of individual medical professions. As a result, legal solutions may facilitate or impede the establishment of interprofessional relations. Although pharmacists, in our study, point to the lack of legal solutions regulating collaboration with doctors, there is a possibility of change in this area. The government’s project of the Act on the profession of the pharmacist [65], defining the professional role and expanding the competence of pharmacists, is currently being processed in Poland. Some opportunities for the collaboration proposed by our respondents are reflected in the project, e.g., the introduction of a clinical pharmacy service that would require collaboration with a physician to determine the patient’s pharmacotherapy [65].

Nevertheless, regulations alone are not enough to build favorable attitudes towards collaboration aimed at improving patient outcomes. The respondents indicated the lack of appropriate training on the topic and presented openness toward implementing joint training sessions as part of post-graduate workplace-based interprofessional education (IPE). In the light of this and previous studies [66, 67], enabling *two or more professions to learn with, from and about each other* while maintaining the context of the workplace seems to be an opportunity to raise PBC and, consequently, the intention to implement the developed cooperative behavior into everyday practice.

### Limitation

We acknowledge that this study has limitations. First, the use of qualitative methodology does not allow to conclude for the entire study population. However, the qualitative approach allowed us to collect in-depth information on the attitudes, opinions, and experiences of pharmacists and physicians involving interprofessional collaboration. Moreover, the final number of respondents was determined based on data saturation to ensure the quality of the study. Next, the researcher’s bias cannot be excluded due to their own opinions on this subject. However, researchers with different backgrounds (pharmacist and physician) were involved in data analysis. Researcher triangulation enabled the reduction of the subjectivity of data analysis and gaining a different perspective on it. Finally, physicians and pharmacists participating in the study could be more open to collaboration. To be able to generalize its perception by doctors and pharmacists, we plan to conduct quantitative research on representative groups based on the results of this study.

The multitude of elements potentially affecting the intention of establishing collaboration suggests the need to develop a quantitative questionnaire allowing for precise determination of the impact of individual elements and adjusting the designed intervention solutions to the needs of individual participants or populations.

## Conclusion

Through the use of Ajzen’s TPB, the study analyzed the way physicians and pharmacists perceive IPC. Although current collaboration between them is limited, their positive attitudes towards collaboration may foster their intention to establish a professional partnership. Doctors and pharmacists present openness to working together and define a pharmacist’s role in the medical team. However, their subjective norms and perceived behavioral control might impede the process and reduce the willingness to collaborate. Respondents raised concerns related to limited awareness of pharmacist’s competencies and mistrust of their intentions among doctors. The lack of pressure from politicians and healthcare decision-makers and a lack of leadership in this field constitute a problem. The respondents also demonstrated low self-confidence in establishing the partnership resulting from insufficient interprofessional competencies. Finally, barriers such as the lack of time, medical staff, or previous negative experiences were also observed.

## Data Availability

Not applicable.

## Abbreviations

ATB: Attitudes towards the behavior
IPC: Interprofessional care
IPE: Interprofessional Education
IRB: Institutional Review Board
PBC: Perceived behavioral control
SN: Subjective norms
TPB: Ajzen’s theory of planned behavior
